# Mitigating Effects of *Rosmarinus officinalis* Essential Oil and Sugar Beet Pulp on Immune Response and Growth Performance of Heat-Stressed Lambs

**DOI:** 10.3390/ani15152241

**Published:** 2025-07-30

**Authors:** Maria Giovanna Ciliberti, Rosaria Marino, Mariangela Caroprese, Cristina Stango, Agostino Sevi, Marzia Albenzio

**Affiliations:** Department of Agriculture, Food, Natural Resources and Engineering (DAFNE), University of Foggia, Via Napoli, 25, 71121 Foggia, Italy; rosaria.marino@unifg.it (R.M.); mariangela.caroprese@unifg.it (M.C.); cristina.stango@unifg.it (C.S.); agostino.sevi@unifg.it (A.S.); marzia.albenzio@unifg.it (M.A.)

**Keywords:** high ambient temperature, feed additives, immune response, lambs

## Abstract

Dietary supplementation under high ambient temperatures can be considered crucial for supporting immune responses in livestock. In the present paper, *Rosmarinus officinalis* essential oil (REO) alone and in combination with dried sugar beet pulp (REO + B) was included in the diet of lambs during the fattening period under heat stress conditions. Environmental conditions and physiological and growth parameters of lambs were monitored throughout the trial. Plasma samples were collected to evaluate cytokine secretion (IL-1β, IL-6, and IL-10). Notably, REO inclusion reduced rectal temperature and respiration rate while increasing pro-inflammatory cytokines (IL-1β and IL-6) at the same time, suggesting an enhanced immune response without compromising growth performance.

## Simple Summary

Recently, essential oils have been widely used as feed additives to improve the sustainability of ruminant production by reducing methane production and enhancing overall health. The aim of the present paper was to evaluate *Rosmarinus officinalis* essential oil (EO) and the combination of *R. officinalis* EO and dried sugar beet pulp addition on the immune profile in terms of cytokine secretion and growth performance of heat-stressed fattening lambs. In this context, a partial substitution of feed with by-products may represent a good strategy for improving livestock sustainability and reducing feed cost. Additionally, EO could support lamb thermotolerance and immune response.

## 1. Introduction

Stressful conditions have a deleterious impact on animal welfare, productive performance, and meat quality of growing lambs [1]. Heat stress (HS) represents a hard challenge for livestock systems, and it impacts the production, reproduction, and health of animals; different strategies are being used to mitigate HS in livestock [2,3,4,5,6,7]. A recent study has demonstrated that HS directly impacts the incidence of cutting dark or pale, soft, and exudative meats in lambs [8,9]. Breeding programs focused on animals and well-adapted to extreme environmental conditions are essential to guarantee food security in the future [10]. In this regard, the most popular sheep breeds used for meat production, such as Texel (Netherlands), Suffolk (UK), and Ile de France (French), exhibit low heat tolerance compared to breeds adapted to the semiarid tropics, such as Santa Inês and Morada Nova [11]. In response to HS, animals activate a cascade of physiological and immunological processes, including the release of pro-inflammatory cytokines such as interleukin-1β (IL-1β) and interleukin-6 (IL-6), alongside anti-inflammatory mediators like interleukin-10 (IL-10) [12]. Modulating the inflammatory responses through dietary strategies has emerged as a promising tool for enhancing resilience to HS [13]. Several essential oils (EOs), such as oregano, thyme, eucalyptus, and garlic, have been found to modulate rumen fermentation and improve animal health and welfare [14]. However, the specific immunomodulatory effects of EOs in ruminants under HS remain underexplored, especially in in vivo studies. In addition, some inconsistencies regarding the efficacy of EOs still exist in animals, largely due to the variety of their chemical composition and the influence of several factors related to animal health, welfare, and farm management, such as infection status, nutritional condition, environmental factors, and, in particular, diet composition [15]. Indeed, as reported by Oh et al. [16], phytonutrients can regulate both adaptive and innate immune responses, depending on the immune challenge status of the experimental animals. Our previous in vitro study using a sheep model of inflammation, in which peripheral blood mononuclear cells (PBMCs) were stimulated with LPS and ConA, demonstrated that *Rosmarinus officinalis*, *Mentha* × *piperita*, and *Lavandula angustifolia* EOs modulate both cell proliferation and cytokine secretion, with EO-specific patterns of response [17].

Moreover, in addition to EO feed inclusion, recently it has become a common practice to partially replace conventional feed with agricultural by-products in order to reduce feeding costs and improve livestock sustainability [18,19,20,21,22]. Among these, pectin-rich feedstuffs such as sugar beet pulp (SBP), a fiber-rich by-product of the sugar industry, are of particular interest. Several studies [23,24] suggest that partially substituting cereal grains with SBP can help maintain rumen health and prevent digestive disorders in fattening lambs; however, no information on immune profile, including cytokine secretion, has been reported. Given the EO and SBP roles in rumen health and their potential to modulate gut microbiota and immunity, we have hypothesized that *R. officinalis* EO (REO) could sustain the immune and growth performance of growing lambs subjected to HS, and the combination of REO with SBP, as a partial replacer of concentrate feed formulation, may represent a sustainable and improved feed formulation aimed at reducing feed cost and feed-food competition and synergistically enhancing the immune and physiological responses of growing lambs to HS. Based on the previous statements, the objective of this study was to examine the effect of two experimental diets containing *R. officinalis* EO microencapsulated into pelleted feed and the partial substitution of feed with beet pulp on physiological responses, immune responses, and growth and carcass performance of lambs exposed to HS.

## 2. Materials and Methods

### 2.1. Animals and Experimental Conditions

The experimental design and all animal procedures were developed in compliance with the Foggia University Institutional Animal Care and Use Committee (protocol number 002-2023). The experiment was conducted during the summer (from 25 June to 29 July) of 2024 in a sheep farm located in the Gargano National Park, which is 60 km north–west of Foggia (Southern Italy), with an elevation of about 250 m above sea level. A total of 30 Texel male lambs, with an average body weight of 25.85 ± 0.66 Kg at the beginning of the trial, were enrolled in this experiment. Lambs were randomly allotted to the following three dietary groups: a control group (CON) fed with pelleted concentrate (1 Kg/lamb/d, Euro Commerce Srl, Italy); an *R. officinalis* EO feed group fed with pelleted concentrate with lipid-based microencapsulated *R. officinalis* EO (REO 0.87% on DM, GreenVet, Forlì, Italy); and a group fed with lipid-based microencapsulated *R. officinalis* EO (0.87% on DM) and pelleted concentrate partially substituted with dried SBP (35.79% on DM) (REO + B). Lipid-based microencapsulation was applied to preserve functional properties of thermosensitive and/or volatile compounds, such as EOs, during the pelleting procedure. Moreover, it was essential to ensure rumen bypass and deliver active compounds to specific target sites, such as the intestine, thereby enabling controlled and site-specific release [25].

All animals received water and oat hay ad libitum. Ingredients and chemical composition of the experimental diets are shown in Table 1. The dietary experiment lasted 35 days (a 7-day adaptation period to the diet followed by 28 days of experimental procedures), in which lambs were housed in individual pens equipped with water dispensers.

Throughout this trial, meteorological parameters, including ambient temperature (T, °C) and relative humidity (RH, %), were continuously monitored by using thermo-hygrographs (TIG2-TH, LSI) positioned 1.5 m above the floor. These data were then used to calculate the temperature–humidity index (THI) according to the following formula proposed by Kelly and Bond [27]:

THI = (1.8 × Tdb + 32) − (0.55 − 0.0055 × RH) × (1.8 × Tdb − 26)

where Tdb is the dry bulb temperature and RH is the relative humidity.

### 2.2. Physiological Parameters

Respiration rate (RR) and rectal temperature (RT) were measured weekly. RR was assessed by counting flank movements over a 20-s period, then converting the count to breaths per minute. Observations were made when the lambs were calm, and the number of waist undulations over one minute was recorded. Three measurements were taken for each lamb, and the average was used as the RR. Rectal temperature was measured using a Vicks Speed Read digital clinical veterinary thermometer. The glass probe was disinfected with an alcohol swab and inserted approximately two-thirds of its length into the rectum. Each RT measurement was repeated three times per lamb, and the average was recorded.

### 2.3. Blood Sampling and Immune Profiles

Blood samples (7 mL) were collected by jugular venipuncture at +7, +21, and +35 days at 7 a.m. before feeding using vacutainer tubes containing sodium heparin as an anticoagulant. Blood samples were immediately centrifuged (1500 g for 15 min at 25 °C), then plasma samples were collected and immediately frozen at −20 °C up until an ELISA test was performed.

Cytokine profile was measured in terms of IL-6, IL-1β, and IL-10 using a sandwich ELISA. For IL-6, IL-1β determination followed the procedure reported in Ciliberti et al. [28]. Briefly, specific mouse monoclonal antibodies against ovine IL-6 (Clone 4B6) and ovine IL-1β (Clone 1D4) were used as capture antibodies (Serotec Ltd., Kidlington, UK). Rabbit polyclonal anti-sheep IL-6 and IL-1β antibodies (Serotec Ltd., Kidlington, UK) were added as detection reagents. All incubations were performed at 37 °C. The concentration was expressed in nanograms per milliliter (ng/mL) by reading the sample against a standard curve built with serial dilutions of recombinant ovine IL-6 (Cusabio Biotech Co., Wuhan, China) and recombinant ovine IL-1β (Kingfisher Biotech Inc., St. Paul, MN, USA).

Secretion of IL-10 was measured in plasma by following Kwong et al.’s [29] procedure, which contains some modifications as reported in Ciliberti et al. [28]. The sandwich ELISA was represented by a monoclonal antibody against bovine IL-10 (Clone CC318, Serotec Ltd., Kidlington, UK) and a biotinylated monoclonal antibody (Clone CC320, Serotec Ltd., Kidlington, UK). Streptavidin–horseradish peroxidase (HRP) (1:500, AbD Serotec) was added as a secondary antibody. IL-10 levels were expressed in nanograms per milliliter (ng/mL). All plates were read at 450 nm using a Power Wave XS microplate spectrophotometer (Biotek Instruments, Winooski, VT, USA).

### 2.4. Growth Performance and Carcass Characteristics

All lambs were individually weighed at weekly intervals in order to estimate the daily gain (DG, Kg/day) and the feed efficiency (FE, Kg DM/daily gain). Individual dry matter intake (DMI) was calculated daily as the difference between the amount of feed offered and the amount of feed refused. At the end of the experiment, animals were fasted for 12 h, weighed, and transported to a local slaughterhouse where they were slaughtered, according to industrial routines used in Italy and the EU rule No. 1099/2009 [30]. After slaughter, the hot carcass weight was recorded and the dressing percentage calculated. Ultimate pH was measured at 24 h using a portable pH meter equipped with a glass electrode and an automatic temperature compensator (CAT) probe (Hanna Instruments, Woonsocket, RI, USA) inserted into the *longissimus thoracis* and *lumborum* muscle between the 12th and 13th ribs.

### 2.5. Statistical Analyses

Data were verified for normal distribution with the Shapiro–Wilk test [31] and then analyzed using the mixed ANOVA in SAS (SAS University Edition, Version 9.4). Data on growth performance and carcass characteristics were subjected to a one-way ANOVA with diet as a fixed effect. For physiological and immune items, the model included dietary treatment (D), time of sampling (T), and their interaction (D*T) as fixed effects. A Tukey post hoc test was applied to adjust for multiple comparisons. Statistical significance was set at *p* < 0.05. Pearson correlation analysis was performed for cytokines measured in the plasma.

## 3. Results and Discussion

### 3.1. Meteorological Data

**Figure 1:**
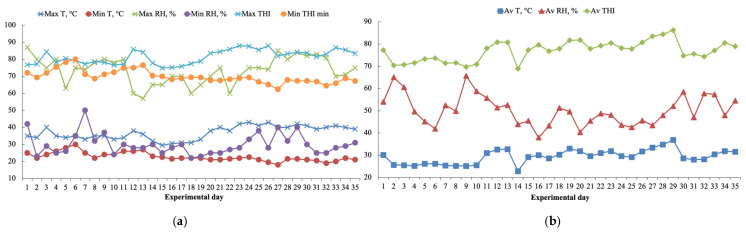
Maximum (max) and minimum (min) temperature (**a**), average (**b**) environmental temperature (T, °C), relative humidity (RH, %), and temperature–humidity index (THI) measured by the Kelly and Bond formula [27] and recorded throughout the experimental days.

The temperature–humidity index (THI) is the most frequently used index for assessing the level of HS in livestock [32]. Exposure to a THI level over 80 with a maximum ambient temperature over 30 °C induces HS in sheep [33], thereby increasing body temperature and activating physiological and behavioral responses that result in serious risks to livestock welfare and health [34]. During the experimental trial, mean daily ambient temperatures ranged from 23 °C to 33 °C, and mean daily relative humidity ranged between 42% and 66%. As a consequence, mean daily THI varied from 72.6 to 80.6 throughout the trial (Figure 1a). Maximum ambient temperatures were reached between the period of 21 days to 35 days, ranging between 43 °C and 42 °C, with the maximum daily THI exceeding 80 (84.4 and 86.2, respectively, Figure 1b). Based on previous statements, growing lambs were exposed to moderate HS conditions (≤82 THI ≤ 84) on the first 7 days and then to severe HS conditions (85 ≤ THI < 86) until the end of the trial.

### 3.2. Respiration Rate and Rectal Temperature

**Figure 2:**
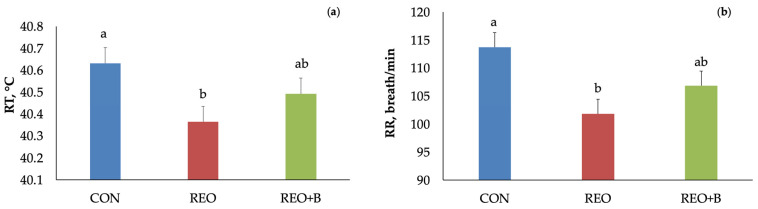
Average of (**a**) rectal temperature (RT) and (**b**) respiration rate (RR) measured in lambs fed with pelleted concentrate (CON), with pelleted concentrate containing microencapsulated *R. officinalis* EO (REO), and with pelleted concentrate containing microencapsulated *R. officinalis* EO and dried sugar beet pulp (REO + B). Bars with different letters are significantly different at *p* < 0.05.

Physiological indicators, such as RR, heart rate, and RT, exhibit a prompt reaction to HS [35], thus serving as effective measures of animal comfort or discomfort. Indeed, the RR and RT are widely used to assess physiological adaptation to HS in small ruminants [36]. In Table 2, data on RR and RT recorded throughout the experiment are shown. The interaction effect between dietary treatment and time did not significantly affect RR values. While, on average, RR was significantly influenced by time of experiment (*p* < 0.001) and dietary treatment (*p* < 0.01, Figure 2a). Lambs that received feed with microencapsulated *R. officinalis* EO showed lower RR than the CON and REO + B groups, supporting a role of *R. officinalis* EO in mitigating the physiological response to heat stress. On average, at +14 days, the lowest value of RR (93.69 ± 3.00) was recorded. Silanikove [36] reported that RR classification in sheep was 40–60 breaths/min for low stress, 60–80 breaths/min for medium-high stress, 80–120 breaths/min indicated HS, and values above 200 breaths/min indicated severe stress. Notably, lambs in the REO and REO + B groups exhibited RR values within the 85–114 breaths/min range, while the CON group showed higher rates, exceeding 120 breaths/min (101.75 ± 5.2 on day 14 and 122.50 ± 5.2 on day 21).

Rectal temperature was also significantly affected by diet (*p* < 0.05, Figure 2b) and time of experiment (*p* < 0.05). On average, the REO group showed lower RT than the CON and REO + B groups. Over time, RT decreased from +7 and +14 days to +28 days. RT typically begins to rise above normal at ambient temperatures of 32 °C, with open-mouth panting observed when RT exceeds 40 °C and RH is below 65% [37]. Under normothermic conditions, sheep maintain a body temperature between 38.3 °C and 39.9 °C [34]. In this study, all groups recorded RT above 40 °C, surpassing the physiological thresholds typically reported for sheep under thermoneutral conditions [34,37]. Based on the previous finding, this study supports that REO inclusion was able to mitigate HS, maintaining an RR value of about 100 breaths/min, suggesting a mild heat stress exposition in lambs due to the capacity of reducing RR. Indeed, REO inclusion effectively reduced RT values, suggesting improved thermotolerance, although the values reached were in line with the threshold indicating an HS exposition. This aligns with previous studies indicating that bioactive compounds in *R. officinalis* have antioxidant and vasodilatory properties that support thermoregulation [38]. In this study, animal physiological indicators have shown that *R. officinalis* EO could alleviate the physiological response activated by lambs under HS conditions. Additionally, the use of a partial substitution of conventional feed with sugar beet pulp in combination with microencapsulated *R. officinalis* EO did not produce any synergistic effects or improvements in the physiological responses of growing lambs under HS conditions. These findings are consistent with literature indicating that increased fermentable fiber can raise endogenous heat load, particularly under HS conditions [39,40], confirming that REO inclusion was able to cope with the thermoregulatory effect of high temperature.

### 3.3. Cytokine Secretion

**Figure 3:**
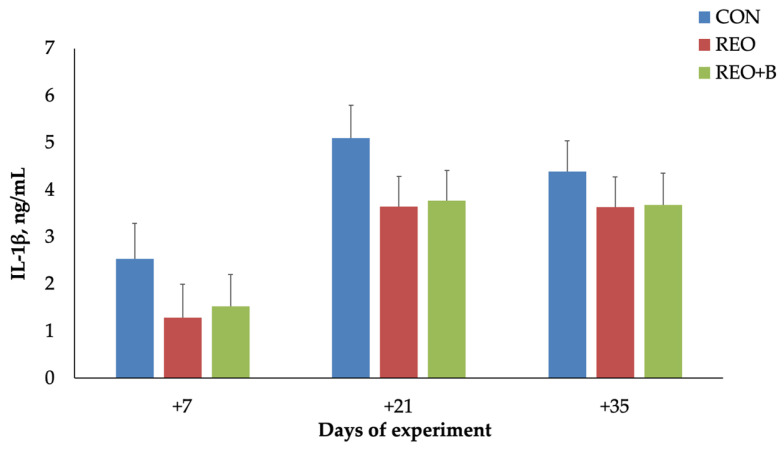
Plasma interleukin-1 beta (IL-1β) secretion (mean ± SEM) of lambs fed with pelleted concentrate (CON), with pelleted concentrate containing microencapsulated *R. officinalis* EO (REO), and with pelleted concentrate containing microencapsulated *R. officinalis* EO and dried sugar beet pulp (REO + B).

Data on cytokine profile showed that IL-1β secretion was significantly affected by dietary treatment (*p* < 0.001), with higher levels observed in both supplemented groups compared to the control (CON) group. No significant effects were found for time or for the interaction between dietary treatment and time (Figure 3). In contrast, IL-6 levels were significantly influenced by dietary treatment (*p* < 0.01), with elevated concentrations observed in the REO and REO + B groups compared to CON. IL-6 secretion also showed a significant time-dependent increase (*p* < 0.001), with levels rising from day 7 to day 21 and continuing to increase through day 35.

**Figure 4:**
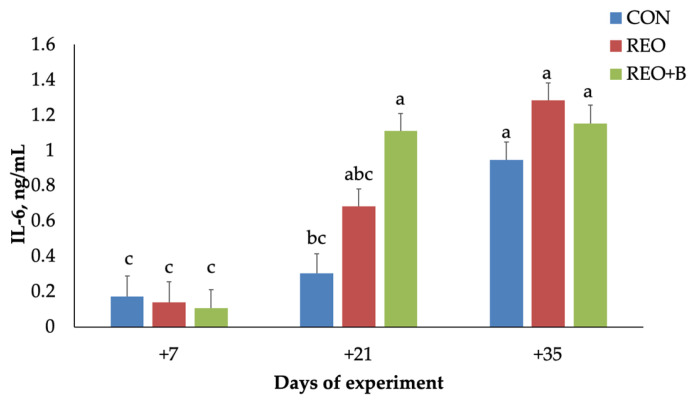
Plasma interleukin-6 (IL-6) secretion (mean ± SEM) of lambs fed with pelleted concentrate (CON), with pelleted concentrate containing microencapsulated *R. officinalis* EO (REO), and with pelleted concentrate containing microencapsulated *R. officinalis* EO and dried sugar beet pulp (REO + B). Bars with different letters are significantly different at *p* < 0.05.

A significant interaction effect between dietary treatment and time of experiment (*p* < 0.001) indicated that IL-6 levels in the REO + B group were higher than those in the CON group on day 21, while the CON group maintained levels similar to baseline (Figure 4).

**Figure 5:**
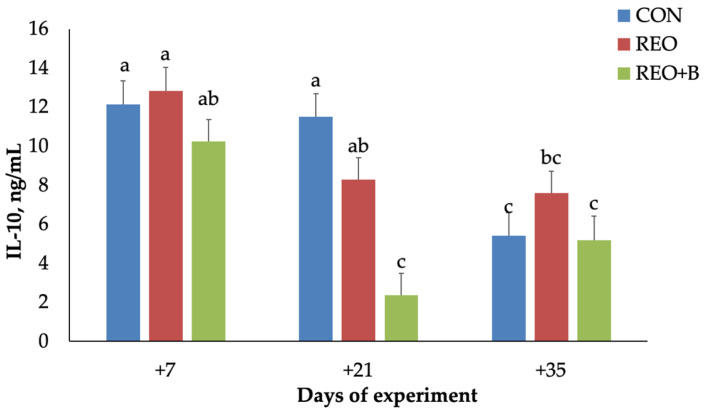
Plasma interleukin-10 (IL-10) secretion (mean ± SEM) of lambs fed with pelleted concentrate (CON), with pelleted concentrate containing microencapsulated *R. officinalis* EO (REO), and with pelleted concentrate containing microencapsulated *R. officinalis* EO and dried sugar beet pulp (REO + B). Bars with different letters are significantly different at *p* < 0.05.

By day 35, all groups exhibited increased plasma IL-6 concentrations. On average, IL-10 levels were significantly lower in the REO + B group compared to both the CON and REO groups (*p* < 0.001), with a notable decrease from day 7 to day 21 (*p* < 0.001). A significant interaction between dietary treatment and time (*p* < 0.01, Figure 5) was also observed. Specifically, the lowest IL-10 concentration was detected in the REO + B group on day 21. Additionally, the REO group showed a significant decrease from day 7 to day 21, while the CON group exhibited lower IL-10 levels on day 35 compared to both earlier time points.

The increase in IL-1β secretion observed in both REO-supplemented groups (REO and REO + B) agreed with previous studies indicating that phytochemicals in *R. officinalis*, such as carnosic acid and rosmarinic acid, may stimulate components of the innate immune response [41]. IL-1β plays a central role in mediating inflammatory responses during thermal stress, and its augmentation may reflect an enhanced immunological response necessary to counteract cellular stress damage. The lack of a time effect further supports the hypothesis that EO bioactive compounds were able to directly stimulate a pro-inflammatory bias independently of HS exposure. In contrast, IL-6 levels were modulated by experimental diet and time of the HS exposition. *R. officinalis* EO-treated groups exhibited significantly elevated IL-6 levels, with the REO + B group showing a pronounced increase on day 21. This is consistent with findings that bioactive compounds from *R. officinalis* can activate IL-6 expression through NF-κB signaling pathways [42]. The observed increase in IL-6 over time is also in line with evidence that prolonged heat exposure may exacerbate systemic inflammation [43]. Moreover, REO combined with beet pulp, as a source of sustainable feed, exerted a synergistic effect in raising the IL-6. This effect may be ascribed to changes in gut microbial activity induced by beet pulp, which has been associated with increased volatile fatty acid production and potential mucosal immune stimulation [43]. The absence of synergistic effects with SBP may stem from its fermentable fiber content, which could exacerbate endogenous heat production under stress.

IL-10, a key anti-inflammatory and modulatory cytokine, was significantly suppressed in the REO + B group, particularly on day 21, suggesting an impaired regulatory response. A similar reduction was reported by Duanmu et al. [44] in HS goats fed high-fiber diets, indicating that feed composed of certain fiber sources may disrupt anti-inflammatory signaling under stress conditions. The absence of a kinetic decline in IL-10 levels in the REO group supports the hypothesis that REO alone may induce a transient pro-inflammatory response without impairing immune regulatory mechanisms. These findings suggest the presence of a moderate balance between immune activation and regulation, which may be disrupted when REO is combined with fermentable fiber.

Pearson correlation analysis reinforces the previous concept. Indeed, a significant negative correlation between IL-6 and IL-10 (r = −0.56, *p* < 0.001 and r = −0.43, *p* = 0.002, respectively) was revealed in both CON and REO groups, which reflects a classical inflammatory counterbalance reported in heat-stressed feedlot lambs [45]. In the REO + B group, the IL-1β and IL-10 were positively correlated (r = 0.33, *p* = 0.02), whereas IL-6 and IL-10 exhibited a strong negative correlation (r = –0.60, *p* < 0.001). Therefore, the REO + B group displayed a more complex inflammatory pattern that could indicate a compensatory anti-inflammatory attempt in the face of heightened inflammation. In a review of Shanmugam et al. [46], the anti-inflammatory effect of 1,8-cineole, one of the major monoterpenoids present in REO, balancing pro-inflammatory and anti-inflammatory cytokine production and controlling acute disease, was discussed.

Collectively, these findings provide new insights into the effects of *R. officinalis* EO and alternative feed strategies on immune function in lambs exposed to thermal stress. While REO alone appears to enhance immune responsiveness without severely compromising regulation, its combination with beet pulp may induce a pro-inflammatory bias. This may have practical implications, especially in thermally challenging environments, where immune overactivation could contribute to subclinical inflammation and reduced productivity [33].

### 3.4. Growth Performance and Carcass Characteristics

Data on growth performance and carcass characteristics of lambs are reported in Table 3. *R. officinalis* EO inclusion alone or in combination with dried SBP did not affect average daily gain, dry matter intake, and feed efficiency of growing lambs.

These findings are consistent with those reported in Norduz [47] and Barbarine lambs [48], where EO inclusion, irrespective of dose and way of administration, did not impact growth performance. Moreover, dry matter intake decreased in all experimental groups during the third week of the trial (*p* < 0.001), coinciding with the highest recorded temperatures. However, the transient reduction in feed intake did not impair daily gain; this may be due to the reduction in the digestion rate, which is responsible for enhancing nutrient absorption and minimizing reduction of feed intake under high ambient temperatures. This adaptive physiological mechanism seems to support previous observations that Texel sheep, although generally considered less heat-tolerant, may activate physiological mechanisms to maintain growth [49].

Slaughtering and carcass weights, dressing percentage, and ultimate pH were unaffected by experimental diets (Table 4). Overall, the final body weights in all groups ranged from 34.89 to 36.1 Kg. While REO did not enhance growth, its ability to maintain performance under stress underscores its value as a resilience-boosting supplement.

Postmortem glycolysis maintained the regular course in all experimental groups with final pH within the acceptable limits for fattening lambs. Therefore, these results suggested that none of the experimental treatments negatively affected meat quality in terms of carcass characteristics and muscle pH evolution after slaughter. Those results confirm previous studies on lambs fed with sugar beet pulp [24], as well as with different types of EOs, such as Cinnamomum zeylanicum [50] and oregano [51]. Notably, to the best of our knowledge, no prior study has investigated the combined use of *R. officinalis* EO and sugar beet pulp as a dietary supplementation strategy in growing lambs.

## 4. Conclusions

In the present study, *R. officinalis* EO inclusion demonstrated beneficial effects on thermo-physiological and immune parameters of fattening lambs without compromising growth performance. However, its combination with SBP did not produce synergistic effects and may even attenuate the benefits observed with REO alone.

In particular, a significant interplay between physiological parameters, respiratory rate and rectal temperature, and cytokine secretion (IL-1β, IL-6, and IL-10) is found, providing insight into the lambs’ overall adaptive capacity under thermal challenge. Interestingly, in the REO group the improvements in physiological response were accompanied by moderate increases in IL-1β and IL-6 pro-inflammatory cytokines. Thus, reflecting a mild inflammatory activation and adaptive immune response to preserve homeostasis during heat exposure, without triggering excessive systemic inflammation. While the stronger inflammatory profile in REO + B lambs, coupled with concomitant higher IL-1β and IL-6 pro-inflammatory cytokines and lower IL-10 levels, was reflected by higher physiological stress markers, as recorded in the control group, indicating a possible synergistic effect between dietary fiber and REO in exacerbating the stress response. The duration and the dosage of dietary treatments of this study highlight the potential for flexible feeding strategies based on the needs of the animals, which is one of the main objectives of the precision feeding approach.

## Figures and Tables

**Table 1:** Ingredients and chemical composition of experimental diets.

	Experimental Diet		
	CON	REO	REO + B
Ingredients, % DM			
Durum wheat and maize bran	22.52	21.68	15.71
Corn grits and flakes	34.64	34.68	14.84
Wheat middlings	12.12	11.27	10.48
Crushed whole-grain beans	6.06	6.07	
Soybean flour	2.60	2.60	
Sunflower flour	2.60	3.47	3.49
Vitamins and Minerals ^1^	4.95	4.95	4.95
Beet molasses	1.73	1.73	1.75
*Rosmarinus officinalis* EO		0.87	0.87
Dried sugar beet pulp			35.79
Chemical composition, % DM			
Crude protein	13.95	13.8	13.65
Ether extract	3.5	4.03	3.3
Crude fiber	6.7	6.38	6
Ash	7.5	7.04	6.5
Lysine	0.38	0.38	0.25
Methionine	0.15	0.16	0.14
ME (MJ/Kg DM) ^2^	11.55	11.75	11.68
*R. officinalis* EO composition ^3^, %			
1,8-Cineole		35.43	
Camphor		19.6	
β-Pinene		13.09	
α-Pinene		11.8	
Camphene		10.5	
β-Myrcene		3.8	
γ-Terpinene		1.4	
α-Phellandrene		2.2	
Borneol		1.7	
Verbenone		0.48	

CON = control; REO = pelleted concentrate containing microencapsulated *R. officinalis* EO; REO + B = pelleted concentrate containing microencapsulated *R. officinalis* EO and dried sugar beet pulp (REO + B). ^1^ Ingredients: vit. A, 2000,000 IU; vit. D, 400,000 IU; vit. E, 50 mg; vit. PP, 8500 mg; vit. B1, 112 mg; vit. B2, 112 mg; vit. B6, 80 mg; vit. B12, 1 mg; d-pantotenic acid, 2400 mg; choline, 15,000 mg; iron, 150 mg; manganese, 800 mg; zinc, 2200 mg; cobalt, 8 mg; iodine, 30 mg; selenium, 5 mg; molibden, 10 mg. ^2^ ME (MJ/Kg DM = metabolizable energy (ME) per kilogram of dry matter [26]. ^3^ Limit quantitation to 0.01%, using the method GC-FID.

**Table 2:** Effect of experimental dietary treatment on lambs’ rectal temperature (RT) and respiration rate (RR) throughout the experimental trial (mean ± SEM).

	Days		Experimental Diet				*p*-Value		
			CON	REO	REO + B	SEM	Diet	Time	D*T
RT, °C	+	7	40.63	40.52	40.74	0.14	0.03	0.01	0.80
	+	14	40.76	40.49	40.67				
	+	21	40.60	40.31	40.33				
	+	28	40.54	40.13	40.22				
RR, breath/min	+	7	108.64	111.11	108.89	5.21	0.01	<0.001	0.51
	+	14	101.75	85.33	94.00				
	+	21	122.50	105.33	110.67				
	+	28	122.00	105.44	113.78				

CON = control; REO = pelleted concentrate containing microencapsulated *R. officinalis* EO; REO + B = pelleted concentrate containing microencapsulated *R. officinalis* EO and dried sugar beet pulp (REO + B).

**Table 3:** Effect of experimental dietary treatment on lambs’ delta body weight (BW), daily gain (ADG), dry matter intake (DMI), and feed efficiency (FE) recorded during the experimental period (mean ± SEM).

		Days	Experimental Diet				*p*-Value		
			CON	REO	REO + B	SEM	Diet	Time	D*T
Delta BW, Kg	+	7	3.50	3.78	3.33	0.51	0.38	<0.001	1.00
	+	14	6.38	6.33	6.00				
	+	21	8.00	7.89	7.56				
	+	28	9.88	9.56	9.00				
ADG, Kg/day	+	7	0.18	0.20	0.18	0.02	0.44	0.17	0.93
	+	14	0.24	0.21	0.22				
	+	21	0.23	0.22	0.22				
	+	28	0.23	0.21	0.18				
DMI, Kg/DM	+	7	1.40	1.40	1.40	0.02	0.11	<0.001	0.29
	+	14	1.59	1.59	1.56				
	+	21	1.38	1.38	1.38				
	+	28	1.56	1.49	1.44				
FE, Kg DM/daily gain	+	7	8.27	9.91	8.26	1.18	0.39	0.24	0.96
	+	14	7.84	9.36	7.42				
	+	21	6.81	7.03	6.92				
	+	28	7.71	8.63	8.81				

CON = control; REO = pelleted concentrate containing microencapsulated *R. officinalis* EO; REO + B = pelleted concentrate containing microencapsulated *R. officinalis* EO and dried sugar beet pulp (REO + B).

**Table 4:** Effect of experimental dietary treatment on lambs’ initial and final body weights and carcass characteristics of the experimental groups (mean ± SEM).

	Experimental Diet				
	CON	REO	REO + B	SEM	*p*-Value
Initial body weight, Kg	26.12	25.55	25.89	0.6	0.943
Final body weight, Kg	36.1	35.11	34.89	0.5	0.782
pH, 24 h	5.88	5.78	5.85	0.03	0.085
Carcass weight, Kg	20.67	19.88	19.95	0.41	0.455
Dressing percentage	57.42	56.62	57.18	0.38	0.386

CON = control; REO = pelleted concentrate containing microencapsulated *R. officinalis* EO; REO + B = pelleted concentrate containing microencapsulated *R. officinalis* EO and dried sugar beet pulp (REO + B).

## Data Availability

All data can be obtained by from the corresponding author upon reasonable request.
